# Assessment of the *E-Selectin* rs5361 (561A>C) Polymorphism and Soluble Protein Concentration in Acute Coronary Syndrome: Association with Circulating Levels

**DOI:** 10.1155/2014/158367

**Published:** 2014-07-24

**Authors:** Elena Sandoval-Pinto, Jorge Ramon Padilla-Gutiérrez, Emmanuel Valdes-Alvarado, Ilian Janet García-González, Angelica Valdez-Haro, Jose Francisco Muñoz-Valle, Hector Enrique Flores-Salinas, Fernando Rivas, Yeminia Valle

**Affiliations:** ^1^Centro Universitario de Ciencias de la Salud, UdeG. Sierra Mojada 950, 44340 Guadalajara, JAL, Mexico; ^2^Instituto de Investigación en Ciencias Biomédicas, Centro Universitario de Ciencias de la Salud, Universidad de Guadalajara, Sierra Mojada 950, Edificio Q, Primer Piso, Colonia Independencia, 44340 Guadalajara, JAL, Mexico; ^3^Centro Universitario de Ciencias de la Salud, Universidad de Guadalajara, Sierra Mojada 950, Edificio Q, Primer Piso, Colonia Independencia, 44340 Guadalajara, JAL, Mexico; ^4^IMSS, Centro Médico Nacional de Occidente, Belisario Dominguez 1000, Colonia Independencia, 44340 Guadalajara, JAL, Mexico; ^5^Hospital General de Occidente, Secretaria de Salud Jalisco, Avenida Zoquipan 1050, Colonia Zoquipan, 45170 Zapopan, JAL, Mexico

## Abstract

*Introduction*. The acute coronary syndrome (ACS) is a complex disease where genetic and environmental factors are involved. *E-selectin* gene is a candidate for ACS progression due to its contribution in the inflammatory process and endothelial function. The rs5361 (561A>C) polymorphism in the *E-selectin* gene has been linked to changes in gene expression, affinity for its receptor, and plasmatic levels; therefore it is associated with an increased risk of cardiovascular disease. The aim of this study was to determine the association of the rs5361 polymorphism with ACS and to measure serum levels of soluble E-selectin (sE-selectin). *Materials and Methods*. 283 ACS patients and 205 healthy subjects (HS) from Western Mexico were included. The polymerase chain reaction-restriction fragment length polymorphism was used to determine the rs5361 polymorphism. The sE-selectin levels were measured by enzyme-linked immunosorbent assay. *Results*. Neither genotype nor allele frequencies of the rs5361 polymorphism showed statistical differences between groups. The sE-selectin levels were significantly higher in ACS patients compared to HS (54.58 versus 40.41 ng/ml, *P* = 0.02). The C allele had no effect on sE-selectin levels. *Conclusions*. The rs5361 *E-selectin* gene polymorphism is not a susceptibility marker for ACS in Western Mexico population. However, sE-selectin may be a biological marker of ACS.

## 1. Introduction

Acute coronary syndrome (ACS) is a term that represents a group of clinical entities whose pathophysiological mechanism comprises ischemia or acute coronary insufficiency. In this spectrum unstable angina (UA), acute myocardial infarction (AMI) with and without ST segment (STEMI and NSTEMI, resp.) elevation is included [1].

Ischemic heart disease is a major public health problem, considered one of the leading causes of death worldwide and the third disease in terms of morbidity and costs [2]. In Mexico, ACS has been recognized as the second cause of death by 2010, surpassed only by type 2 diabetes (DM2) [3].

Age is an outcome determinant of ACS. After the age of 40 years, the risk of developing coronary artery disease (CAD) during lifetime is 49% for male and 32% for female with a sex ratio of 3.4 to 1, respectively [4]. The main risk factors of ACS are hypertension (HBP), dyslipidemia, DM2, smoking, positive family history of coronary heart disease, and overweight/obesity [5].

It is known that thrombosis underlies most complications in cardiovascular diseases. Atherosclerosis is a chronic, inflammatory, immune, and metabolic disease beginning in childhood and continues to develop throughout life. It is considered an exaggerated response to the endothelium aggression towards the formation of fat and fibrous lesions in the intima layer of the arteries. The vessel-lumen diameter narrows with a decrease of the blood-flow. The final event is the ischemia as a consequence of the rupture of the plaque with the thrombus formation [6].

Surface adhesion molecules, such as Selectins family, contribute to the development and progression of atherosclerosis. E-selectin is only expressed in activated endothelial cells by proinflammatory cytokines and mediates leukocyte adhesion and platelet leukocyte interaction [7]. Soluble types of these molecules are shedding from activated cells and can be measured in peripheral blood [8]. Many studies have reported a relationship between circulating adhesion molecules levels and coronary artery disease [9–16].

*E-selectin* gene is a plausible candidate for the development of ACS, primarily for its participation in the inflammatory process and endothelial function. The rs5361 (561A>C) polymorphism in the* E-selectin* gene has been associated with changes to ligand affinity, thereby increasing the risk of cardiovascular disease [17].

In this work, we evaluated the association of the rs5361 polymorphism and circulating levels of sE-selectin in ACS patients and healthy subjects (HS) from a Western Mexico population.

## 2. Methods

### 2.1. Subjects

We studied 488 genetically unrelated individuals with the following characteristics.

1. Two hundred and five HS were recruited (82 males and 123 females). They responded to a questionnaire on their medical history and lifestyle characteristics with absence of cardiovascular diseases history as the main exclusion criteria. None had clinical signs of acute or chronic illness or was receiving any treatment.
2. Two hundred and eighty-three patients with ACS (226 males, 57 females) were diagnosed according to the American College of Cardiology (ACC) criteria [6]. Routine biochemical test measures were registered. Classical risk factors, defined according to the ACC, were categorized as present or absent.

All subjects were recruited from the “Hospital de Especialidades del Centro Médico Nacional de Occidente del Instituto Mexicano del Seguro Social (CMNO-IMSS).” Individuals with other diseases such as infectious, cancer, and autoimmune pathologies were not considered.

### 2.2. Ethical Considerations

The study was made in accordance with the Declaration of Helsinki. All patients and subjects accepted to participate and an informed written consent was obtained. Ethical approval was obtained by the Centro Universitario de Ciencias de la Salud, CUCS, UdeG.

### 2.3. Genetic Analysis

Genomic DNA (gDNA) was purified from total leukocytes in peripheral blood, by means of the technique of Miller et al. [18]. gDNA concentration was determined spectrophotometrically at a wavelength of 260 nm (absorbance of nucleic acids) and 280 nm (absorbance of proteins). Once gDNA concentration was obtained, the samples were stored at −20°C until use.

The rs5361 polymorphism was amplified by polymerase chain reaction-restriction fragment length polymorphism (PCR-RFLP) using the following primers sequences: forward, 5′-CCG TAG CTG CCT GTA CCA AT-3′; and reverse, 5′-CAG AGT CGA GTG CTA GTG GA-3′. PCR amplification was carried out in a total volume of 20 *μ*L containing 10 ng/*μ*L of gDNA, 0.08 U/*μ*L of Taq DNA polymerase (Invitrogen, Carlsbad, CA, USA), 1X of buffer, 0.8 pM of each primer, 1.5 mM of MgCl_2_, and 0.1 mM dNTP. The thermocycling conditions had an initial denaturation step of 3 min at 94°C followed by 32 cycles of 30 s each at 94°C, 57°C, and 72°C and the final extension step of 1 min at 72°C. A 249 bp PCR fragment was digested using 5 U of* PstI* enzyme (New England BioLabs, Beverly, MA) in a final volume of 15 *μ*L during 2 h at 37°C. Digestion of the PCR product resulted in 219 + 30-bp (A) or 249-bp (C) fragments. The amplified PCR and digested products were analyzed by electrophoresis in 6% polyacrylamide gels (29 : 1; acrylamide : bisacrylamide) stained with silver nitrate.

### 2.4. Analysis of sE-Selectin Levels

The sE-selectin levels were measured in duplicate using serum samples from ACS patients and HS by enzyme linked immunosorbent assay (ELISA) according to manufacturer specifications (R&D Systems, Minneapolis, MN, USA). The sE-selectin range was 12.5–84 ng/mL and the sensitivity of the assay was 0.09 ng/mL. sE-selectin concentration was calculated using a four-parameter logistic (4-PL) curve fit.

One hundred and thirteen HS were included (67 females and 46 males) with a mean age of 54 years (range 29–84 years). Regarding ACS patients, diagnosis (16 UA, 19 NSTEMI, and 83 patients with STEMI) and variant carriers were also considered, resulting in 118 individuals (45 females and 73 males) with a mean age of 62 years (range 33–87 years).

### 2.5. Statistical Analysis

The statistical analysis was carried out using SPSS statistical package version 21.0, Excel 2010, and GraphPad Prism 6.04 (GraphPad software, CA, USA). The *χ* ^2^ or Fisher's exact test, when applicable, was used to compare discrete variables and to test the Hardy-Weinberg equilibrium. The data for continuous variables were expressed as means ± standard deviation (SD) and mean differences were evaluated by Mann-Whitney* U* test. The odds ratio (OR) was the measure of association. The significance level was *P* < 0.05. In order to rule out the age and gender bias in the measure of sE-selectin levels, a linear regression was applied.

## 3. Results and Discussion

### 3.1. Demographic and Clinical Characteristics

The mean age of ACS patients was 63 ± 11.8 years. The main gender affected was males with at least one risk factor (Table 1). This is consistent with the data from the Mexican Register of ACS, RENASICA II [19], where patients were older than 60 years and 72% were represented by males. British Heart Foundation [20] denotes age as the most powerful independent predictor for ACS, and among males, the risk increases by age. This is noteworthy because according to data of the INEGI [21] there are 10.9 million of older adults (>60) in Mexico and the ischemic heart disease accounts for 16.2% of deaths. Consequently, the mortality rate associated with cardiovascular diseases has risen 46% in the last nine years involving higher costs to health institutions. Particularly myocardial infarction (MI) had a cost of 527 million dollars per year in the Mexican Social Security Institute [22]. In addition, younger populations are increasingly being affected when compared to other regions because the principal risk factors, such as HBP, are more prevalent [23, 24].

Only mean glucose levels were higher than reference values. The triglycerides and cholesterol means were normal because patients were under specific treatment. As expected, cardiac markers were elevated. The most prevalent risk factors were HBP and DM2 (60.7 and 46.3%, resp.). According to RENASICA, HBP/DM2 are markers for mortality accounting for 2.6 times more risk in ACS patients [25]. It is noteworthy to mention that presence of these comorbidities has increased in the Mexican population [3].

Additionally, all these variables were categorized according to dominant genetic model (AA versus AC + CC) without significant differences, ruling out a plausible role of the rs5361 (561A>C) polymorphism in the modification of clinical characteristics related with ACS.

### 3.2. Genetic Contribution

The selectins are transmembrane proteins that have three domains in their extracellular segment (N-terminal Ca^2+^ dependent lectin domain, a single epidermal growth factor-like (EGF-like) domain and 2, 6, or 9 numbers of short consensus repeats) [26]. In humans, E-selectin [27] is expressed in activated endothelial cells and functions as a mediator of leukocyte recruiting at the sites of inflammation.

The identification of genetic variants has allowed improvement of our understanding of pathogenetic diseases. Scarce genome-wide scanning studies exist in relation to ACS. Chiodini and Lewis [28] found that two loci (3q26-27 and 2q34-37) might contain susceptibility genes for CAD. In ACS, Banerjee et al. [29] reported that the ACS heritability is greater when both parents are affected (OR = 5.97, *P* < 0.0001), revealing that genetic studies could have implications in the clinical practice with the opportune identification of the potential risk for individuals as well as an added benefit for pharmacogenomics.

One of the most studied polymorphisms in* E-selectin* gene is the rs5361, which is located in the EGF-like coding region, which causes a variation of an adenine by cytosine at the 561 gene position traducing a serine/arginine (S128R) change in the protein [30]. This variation alters the ligand-binding specificity which leads to a gain of function, amplifying the number of leukocytes that roll and subsequently adhere to the vascular lining [31–33]. Jilma et al. [34] showed that transduced endothelial cells of the 561C allele carriers support significantly more rolling and adhesion of neutrophils and mononuclear cells compared to the wild-type.

Several polymorphisms in* E-selectin* gene have been described. However, the rs5361 polymorphism was selected because its biological impact could be implicated in ACS. In addition, the minor allele frequency (C allele) reported in Mexican population was greater than 5% which allows detecting the variant and making reliable group comparisons [35].

The rs5361 polymorphism has been studied in autoimmune diseases [35, 36], hepatitis [37], HBP [38], venous thrombosis [17], the postoperative MI [39], prognosis for colorectal [40], and breast [41] cancer, as well for severity of atherosclerotic arterial disease [42].

Endothelial dysfunction has been the hallmark of several inflammatory diseases [43]. The integrity of endothelial cell activity is a key factor related with atherosclerosis which is also associated with cardiovascular complications [44, 45]. In addition, the acute inflammatory response plays a pivotal role in ACS demonstrated by higher levels of proinflammatory cytokines and acute phase proteins in the serum of these patients [45]. Together this data supports the participation of E-selectin in these chronic diseases and the importance of further investigation of genetic variability among populations.

In this study, the genotype distributions were in accordance with Hardy-Weinberg equilibrium expectations (Table 2). The minor allele frequency found in controls for C allele was 4%. Neither genotype nor allele frequencies showed statistically significant differences between groups arguing that this polymorphism is not a susceptibility genetic factor for ACS in a Western Mexican population.

**Figure 1:**
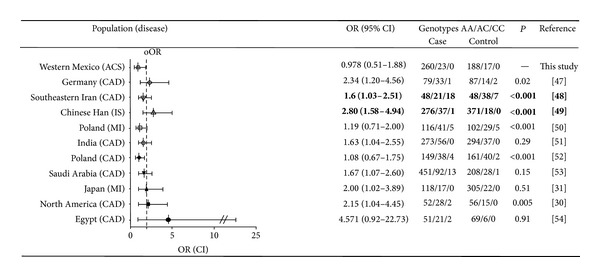
Forest plot of ORs for CAD, MI, and IS in the dominant model (CC + AC versus AA) of the SELE gene A561C polymorphism stratified by ethnicity. oOR: overall OR. Studies where genetic association was significant are highlighted in bold. *P* values for population comparisons were evaluated in healthy subjects (control group). CAD: coronary artery disease; MI: myocardial infarction; IS: ischemic stroke.

The genetic association for the rs5361 polymorphism in ACS has been evaluated in only one study with similar results [46]. Other researchers have evaluated this polymorphism in the context of different cardiovascular diseases (Figure 1). In an ethnical group of China (Han), the carriers of at least one C allele had 2.8 more susceptibility to present ischemic stroke (IS, *P* < 0.001); likewise the dominant genetic model for the rs5361 polymorphism was associated with CAD (OR = 1.6, *P* = 0.04). In a recent meta-analysis, two polymorphisms in the* E-selectin* gene, including the rs5361, have been associated with increased risk of CAD evaluated in Caucasians and Asians. In this study, the C allele was consistently associated with CAD in the codominant and dominant model by ethnicity and grouped (OR > 1.74, *P* < 0.01) [47]. The evaluation of this polymorphism in other entities, such as myocardial infarction, and other population groups (African, American, and European) reveals a lack of genetic association similar to this study. Considering the probability value independently, in the different groups where the rs5361 has been analyzed, the overall association measure was 2.0 (Figure 1) [30, 31, 48–55].

Regarding the allele frequencies of the rs5361 (561A>C) polymorphism in Western Mexico, the genetic distribution was different to those observed in German, Southeastern Iranian, Chinese Han, Polish, and North American populations (*P* < 0.02). These differences can be explained by the genetic structure of Western Mexico mestizos [56].

As can be noticed, extensive data involving CAD exists. CAD is a chronic heart disorder that includes both stable angina and ACS [57]; under the hypothesis that the rs5361 polymorphism could be involved in a particular clinical characteristic of ACS, we constructed an intragroup stratification. The diagnosis and risk factors (DM2, HBP, and DYS) in the ACS group were compared by genotype, allele, and genetic model; however, these comparisons did not reveal a significant difference (data no shown). Nonetheless, as it is stated above, angina and NSTEMI are underrepresented (*n* = 29 and 40 resp.) in our cohort, limiting in part the interpretation of the genetic role of this polymorphism in the ACS type. Further studies are required in order to confirm this lack of association.

### 3.3. sE-Selectin Serum Levels

E-selectin is a surface glycoprotein molecule [58] that supports the rolling of leukocytes on activated endothelial cells and usefully mediates the adhesion of circulating monocytes and lymphocytes to endothelial cells [59, 60]. The regulation of endothelial leukocyte adhesion is a key event in the development of the inflammatory response [43].

Under cellular activation, the soluble isoforms of these adhesion molecules are rapidly shed from the cellular surface [61]. It is hypothesized that the Disintegrin and metalloproteases (ADAMs) members are responsible for shedding the molecules from the cellular surface [62].

Expression induction of vascular selectins (E- and P-selectins) allows recruitment of some immunological cells to inflammatory sites [63]. Previous reports have shown an increase in the selectin-expression of endothelial cells in arteries and a greater interaction of lymphocytes and macrophages during the development of atherosclerotic lesions [64].

In these disorders, the monocytes are key components in the cellular infiltrates [65]. However, chronic expression of E-selectin together with proteolytic shedding from the endothelial surface rises plasma levels of soluble E-selectin; thus these selectins have been associated with inflammatory conditions such septic shock, cardiovascular disease, and DM2 [63].

**Figure 2:**
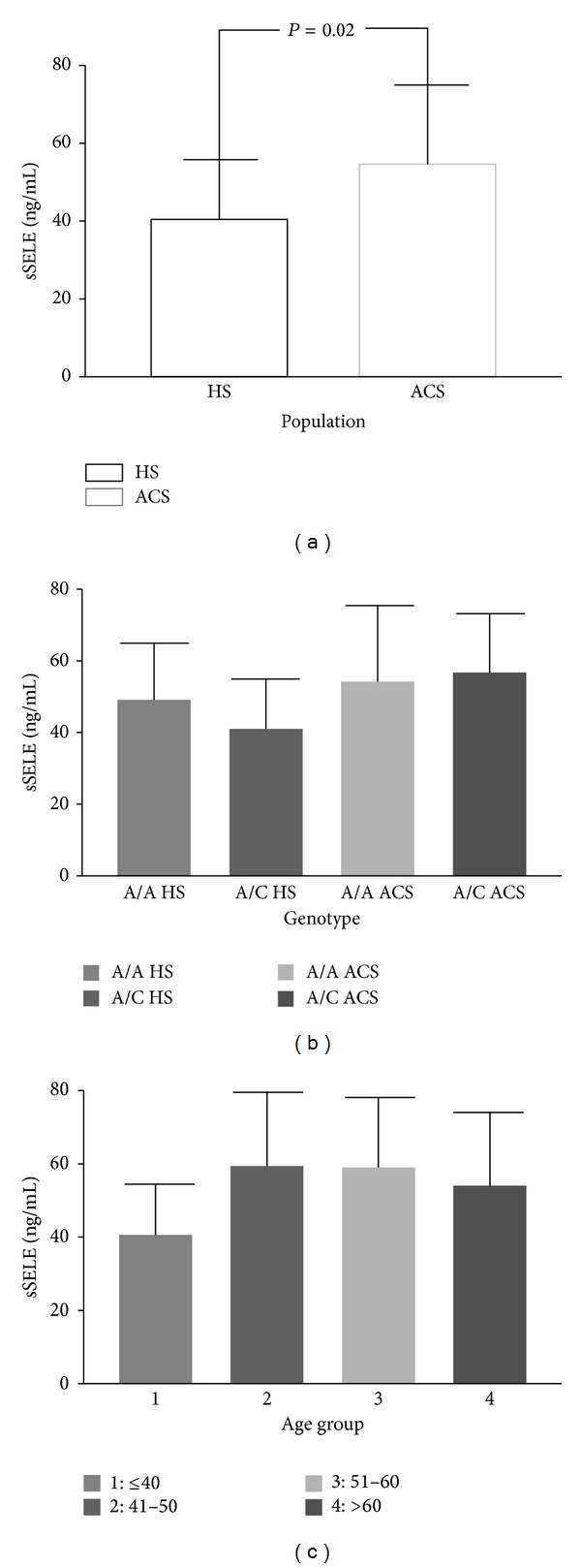
Serum levels comparison of sE-selectin. (a) Comparison of sE-selectin levels in serum of ACS patients (*X* = 54.58 ng/mL; SD = 20.56) and HS (*X* = 40.41 ng/mL; SD = 15.58), (b) comparison of sE-Selectin levels in serum of ACS patients and HS according genotype of the rs5361 (561A>C) polymorphism: ACS patients AA (*X* = 54.19 ng/mL; SD = 21.29); AC (*X* = 56.73 ng/mL; SD = 16.27); HS AA (*X* = 49.13 ng/mL; SD=15.60); AC (*X* = 41.03 ng/mL; SD = 13.82), (c) comparison of sE-selectin levels in serum of HS by age range (1)* X* = 40.58 ng/mL; SD = 13.93; (2)* X* = 59.36 ng/mL; SD = 20.30; (3)* X* = 58.96 ng/mL; SD = 19.30; (4)* X* = 54.05 ng/mL; SD = 20.14. Mean ± standard deviation is shown in graphic representation. Only significant differences are highlighted. ACS: acute coronary syndrome; HS: healthy subjects; sSELE: soluble E-selectin levels. *P*: Mann-Whitney* U* test.

In the present study, serum levels of E-selectin were significantly higher in ACS patients compared to HS (54.58 versus 40.41 ng/mL, *P* = 0.02, Figure 2(a)). These results are consistent with a previous report describing raised sE-selectin levels in patients with ACS [66]. According to ACS type, Macías et al. [67] reported increased levels in patients with UA at admission and ten days later suggesting that sE-selectin could be a marker for unstable angina and might be useful in the differential diagnosis of myocardial infarction. To address this issue, we made soluble level comparisons by ACS type. Even though our sample size was similar to Macías et al. 2003 (16 versus 11), we could not confirm this conclusion since the difference did not reach a statistical significance (data not shown). Some researchers have suggested that soluble levels of E-selectin are gender dependent, being higher in men [68–71]. To test whether age and gender could be introducing bias in our results, the soluble levels of E-selectin were adjusted by age and gender (Table 3) without any significant evidence (*P* > 0.27), reinforcing that higher levels are related with the disease group tested in this study.

Several studies have evaluated sE-selectin and reported significant increase in serum levels in other cardiovascular diseases such as CAD [72–75]. In contrast to the earlier hypothesis that soluble adhesion molecules have an anti-inflammatory role, evidence suggests a unique role for sE-selectin as a proinflammatory and angiogenic mediator [65, 76]. These studies demonstrate that sE-selectin is a potent chemotactic factor for monocytes, and its neutralization leads to a significant decrease in CAD mediated by monocyte migration. Furthermore, their findings suggest that sE-selectin mediates signaling in monocytes through a Src-Ras-MAPK pathway. The inhibition of Src kinase by the Src-specific inhibitor, PP2, abolished sE-selectin mediated monocyte chemotaxis. Thus sE-selectin mediates chemotaxis through the Src pathway and could be a potential target for modulating monocyte recruitment-driven diseases. On the other hand, some investigations have demonstrated that neutrophil exposure to sE-selectin specifically induces actin reorganization, associated with cell polarization without altering cell deformability as was measured by cell transit time in cell filtration assays. Further research also found that sE-selectin specifically potentiates integrin-mediated adhesion [63, 77–79]. These findings, including the present one, reflect the participation of sE-selectin in pathologies that converge in a chronic inflammatory state involving endothelial dysfunction.

Regarding serum levels a correlation between mutational status and E-selectin concentrations was evaluated. However, serum concentrations of E-selectin were similar between carriers and no carriers of C allele (Figure 2(b)) ruling out a genotype-phenotype correlation. Miller et al. [80] also reported that the C allele had no effect on sE-selectin levels evaluated in HS from three ethnic groups (White, African, and South Asian origin). Wu et al. [37] found contrasting results, where C allele carriers had higher sE-selectin levels than those with the A allele (*P* < 0.05), corroborated in HS and chronic hepatitis B patients from Chinese Han population. To the best of our knowledge, there are no published results where the rs5361 (561A>C) polymorphism and serum levels of E-selectin are correlated in ACS. In addition, we stratified sE-selectin levels in HS by age demonstrating no significant differences (Figure 2(c)) which is consistent with an early report [81].

## 4. Conclusion

The rs5361 (561A>C) polymorphism is not a genetic susceptibility factor to ACS. However, the higher serum levels found in patients suggest that sE-selectin could be a biological marker of endothelial activation reflecting its early participation in the acute event in these patients.

## Figures and Tables

**Table 1:** Demographic and clinical characteristics in ACS according to rs5361 (561A>C) *E-selectin* polymorphism following a dominant genetic model.

	561A>C *E-selectin* in ACS			
	All patients *n* = 283 (%)	A/A *n* = 260 (%)	A/C + C/C *n* = 23 (%)	*P* value
	Average ± SE			
Demographics				
Age, years	63 ± 11.8	63.24 ± 11.729	61.13 ± 12.312	0.364
Gender male/female	226/57 (79.9/20.1)	209/51 (80.4/19.6)	17/6 (73.9/26.1)	0.307
Glucose (mg/dL)	161.626 ± 70.74	158.884 ± 67.7846	205.5 ± 136.4716	0.556
Triglycerides (mg/dL)	171.167 ± 41.4894	171.167 ± 41.4894	0 ± 0	—
Cholesterol (mg/dL)	181 ± 53.075	181 ± 53.075	0 ± 0	—
CK (IU/L)	924.71 ± 1509.328	910.45 ± 1540.689	1093.07 ± 1097.892	0.099
CK-MB (IU/L)	110.37 ± 167.070	109.93 ± 170.653	116.05 ± 114.381	0.265
Troponin T (ng/mL)	7.2212 ± 14.10267	6.7461 ± 14.30236	12.3385 ± 10.89742	0.118
Diagnosis				
UA	29 (10.2)	26 (10.0)	3 (13.0)	0.427
STEMI	214 (75.6)	200 (76.9)	14 (60.9)	0.076
NSTEMI	40 (14.1)	34 (13.1)	6 (26.1)	0.86
Risk factor				
Obesity	112 (39.6)	100 (38.5)	12 (52.2)	0.143
DM2	131 (39.9)	123 (47.3)	8 (34.8)	0.175
DYS	117 (41.3)	107 (41.2)	10 (43.5)	0.497
HBP	172 (60.8)	159 (61.2)	13 (56.5)	0.411
Treatment				
AA	224 (79.2)	208 (80.0)	16 (69.6)	—
ACE inhibitors	147 (51.9)	136 (52.3)	11 (47.8)	—
AnA	271 (95.8)	249 (95.8)	22 (95.7)	—
ARB	33 (11.7)	31 (11.9)	2 (8.7)	—
CCB	4 (1.4)	4 (1.5)	0 (0)	—
Antiarrhythmics	5 (1.8)	4 (1.5)	1 (4.3)	—
Diuretic	66 (23.3)	60 (23.1)	5 (21.7)	—
Antilipid therapy	233 (82.3)	213 (81.9)	20 (87.0)	—

Quantitative and qualitative data were evaluated by Mann-Whitney *U* test and exact test, respectively. CK: creatine phosphokinase; CK-MB: creatine phosphokinase MB; UA: unstable angina; STEMI: ST elevation myocardial infarction; NSTEMI: non-ST elevation myocardial infarction; DM2: type 2 diabetes mellitus; DYS: dyslipidemia; HBP: high blood pressure; AA: antithrombin agents; AnA: antiplatelet agents; ARB: angiotensin II receptor blockers; CCB: calcium channel blockers; SE: standard error.

**Table 2:** Allele and genotype distribution of rs5361 (561A>C) *E-selectin* polymorphism by group.

	HS^a^ *n* (%)	ACS *n* (%)	OR (CI)	*P*
Genotype				
A/A	188 (91.7)	260 (91.9)	—	—
A/C	17 (8.3)	23 (8.1)	0.978 (0.508–1.882)	0.948
C/C	0 (0)	0 (0)	0.724 (0.014–36.631)	1.000
Allele				
A	393 (95.9)	543 (96.0)	—	—
C	17 (4.1)	23 (4.0)	1.021 (0.538–1.937)	0.949
Dominant				
A/A	188 (91.7)	260 (91.9)		
A/C + C/C	17 (8.3)	23 (8.1)	0.978 (0.508–1.882)	0.948
Recessive				
A/A + A/C	205 (100)	283 (100)	—	—
C/C	0 (0)	0 (0)	0.725 (0.014–36.681)	1.000

HS: healthy subjects; ACS: acute coronary syndrome; OR: odds ratio, *n*: sample size; CI: confidence interval; *P*: probability value using exact test. *P* = 0.5 for Hardy-Weinberg equilibrium.

**Table 3:** Linear regression analysis by age and gender.

	Standardized coefficient	Confidence interval		P value
	Beta	Lower limit	Upper limit	
Age	0.110	−3.356	8.925	0.270
Gender	0.088	−16.225	3.074	0.371

Dependent variable: sE-selectin levels.
